# Editorial: Sustainable management of *Tuta absoluta*

**DOI:** 10.3389/fpls.2026.1788650

**Published:** 2026-02-23

**Authors:** Ahmad A. Omar, Inusa Jacob Ajene, Hafiz Muhammad Usman Aslam, Fathiya Mbarak Khamis

**Affiliations:** 1 Institute of Food and Agricultural Sciences, Citrus Research and Education Center, Horticultural Sciences Department, University of Florida, Lake Alfred, FL, United States; 2 Faculty of Agriculture, Biochemistry Department, Zagazig University, Zagazig, Egypt; 3 International Centre of Insect Physiology and Ecology (icipe), Nairobi, Kenya; 4 College of Tropical Agriculture and Human Resilience, University of Hawai’i at Manoa, Komohana Research and Extension Center, Hilo, HI, United States; 5 Department of Plant Pathology, San Luis Valley Research Center, Colorado State University, Fort Collins, CO, United States; 6 Department of Zoology and Entomology, University of Pretoria, Hatfield, South Africa

## Introduction

The tomato leafminer, *Tuta absoluta* (Meyrick), remains one of the most devastating global threats to tomato production. Its rapid invasion across continents, exceptional reproductive potential, cryptic foliar-mining behavior, and accelerated evolution of insecticide resistance have significantly complicated management efforts. The persistent failure of conventional chemical strategies underscores an urgent need for integrated solutions that combine molecular innovation, ecological engineering, and rigorous resistance monitoring. This editorial synthesizes key insights from recent contributions by Ullah et al., Bavithra et al., and Adams et al., which collectively outline a transformative, multi-dimensional framework for sustainable *T. absoluta* suppression.

## Global resistance surveillance: alarming trends and strategic implications

The comprehensive review by Bavithra et al. provides a critical global overview of baseline susceptibility patterns and resistance trajectories in T*. absoluta*. Their analysis documents 59 confirmed resistance cases worldwide, spanning virtually all major insecticide classes, including organophosphates, pyrethroids, neonicotinoids, diamides, avermectins, spinosyns, and growth regulators. In several regions, notably Brazil and Southern Europe, resistance to diamides has escalated to extremely high levels exceeding 200,000-fold, emphasizing the magnitude of the crisis. The authors highlight the polygenic nature of evolved resistance, governed by both metabolic detoxification mechanisms (overexpression of P450s, esterases, GSTs) and target-site mutations in sodium channels and ryanodine receptors.

The review further underscores how frequent applications, lack of rotation among Insecticide Resistance Action Committee (IRAC) modes of action, and the pest’s short generation intervals jointly accelerate resistance. Bavithra et al. advocate for strengthening baseline susceptibility datasets, adopting 30-day mode-of-action rotation windows, and integrating biological and cultural tactics to minimize reliance on insecticides. Their work provides an essential foundation for re-designing national and regional resistance-management protocols.

Overall, the article contributes a foundational reference for policymakers, agronomists, and researchers seeking sustainable long-term control of *T. absoluta*.

## Molecular disruption of resistance: RNAi as a next-generation tool

While global surveillance identifies the scale of the challenge, Ullah et al. advance a promising molecular approach to reverse resistance phenotypes. Their research isolates two key detoxification-related genes, CYP9A306 and CYB5R, which are significantly upregulated in cyantraniliprole-resistant *T. absoluta* populations. Using a nanocarrier-mediated delivery system based on star polycation (SPc), the authors successfully introduced dsRNA targeting these genes, achieving 57–59% suppression of transcript levels, a substantial reduction in cytochrome P450 enzyme activity, and a remarkable 73–77% increase in larval mortality following cyantraniliprole exposure.

This study demonstrates that RNA interference (RNAi), when supported by protective nanocarriers that enhance dsRNA stability and cellular uptake, can effectively dismantle specific metabolic resistance pathways. By bypassing cross-resistance challenges commonly observed with chemical insecticides, RNAi offers a precision-based strategy aligned with sustainable pest-management goals. These findings open new avenues for the development of RNAi-based biopesticides and gene-targeted resistance-mitigation tools.

Overall, the research provides in-depth mechanistic insight into cyantraniliprole resistance, while offering a promising technological solution, nanocarrier-mediated RNAi, to enhance susceptibility in resistant pest populations and support sustainable pest control.

## Chemical ecology and habitat manipulation: leveraging volatile-mediated interactions

The ecological dimension of sustainable management is enriched by the work of Adams et al., who explore how volatiles emitted by non-host Asteraceae plants influence the tritrophic interactions among cultivated tomato, *T. absoluta*, and its zoophytophagous predator *Nesidiocoris tenuis*. Their behavioral assays reveal that T. absoluta females are reliably attracted to host-plant monoterpenes, notably β-phellandrene, d-2-carene, α-phellandrene, p-cymene, and terpinolene, while strongly avoiding sesquiterpene-rich volatiles emitted by marigold (*Tagetes minuta*), blackjack (*Bidens pilosa*), and even wild tomato.

Importantly, the mirid predator *N. tenuis* showed no aversion to these repellent sesquiterpenes and responded positively to low concentrations of host monoterpenes. This presents a strategic ecological opportunity: companion cropping with Asteraceae plants can suppress pest colonization without disrupting natural enemy efficacy. The identification of specific attractant and repellent compounds further suggests the potential to develop synthetic kairomone/allomone blends to support pest monitoring or behavioral disruption.

Overall, Adams et al. provide a mechanistic understanding of how non-host plant volatiles can be leveraged to disrupt pest behavior while preserving predator effectiveness, an important step toward more sustainable, chemical-free management of Tuta absoluta in tomato systems.

## A unified, future-oriented strategy for *Tuta absoluta* management

Collectively, these three studies showcase how integrating global resistance intelligence, molecular precision tools, and chemical-ecology-based habitat manipulation can redefine management strategies for *T. absoluta*. The implications are profound:

- From reactive to predictive resistance management: Incorporating baseline susceptibility data and monitoring resistance mechanisms can inform rational, region-specific insecticide rotations.
- From chemical dependence to molecular intervention: RNAi enables targeted suppression of resistance genes, enhancing insecticide efficacy while reducing active ingredient inputs.
- From monoculture vulnerability to ecological fortification: Companion planting and volatile-based strategies can support ecological resilience, decrease pest pressure, and complement biological control.

A forward-looking research agenda should prioritize field validation of nanocarrier-mediated RNAi, quantifying the long-term ecological compatibility of Asteraceae companion plants, and developing decision-support frameworks that integrate resistance monitoring with chemical-ecology tools.

## Conclusion

The increasing severity of *T. absoluta* infestations, coupled with the global proliferation of insecticide resistance, demands a multidimensional paradigm shift. The studies highlighted in this editorial collectively provide a roadmap toward sustainable, science-driven pest management, grounded in molecular biology, ecological resilience, and informed resistance stewardship. In an era of rapid global change and escalating agricultural pressures, such integrative approaches will be essential for safeguarding tomato production and strengthening global food security.

